# A description of community-based participatory research of hypertension awareness, prevention and treatment in a district of Matabeleland South Province, Zimbabwe

**DOI:** 10.4102/phcfm.v11i1.1839

**Published:** 2019-01-31

**Authors:** Pugie T. Chimberengwa, Mergan Naidoo

**Affiliations:** 1 Discipline of Public Health Medicine, University of KwaZulu-Natal, South Africa

## Abstract

**Background:**

Hypertension is an important worldwide public health challenge because of its high prevalence and concomitant risks of cardiovascular and kidney diseases. The risk factors for hypertension are well known, and screening, diagnosis and treatment of hypertension have been well researched. However, this knowledge has not been translated into community practice as there remains a huge knowledge gap between the academics, health workers and the communities. There is need for community participation in developing and implementation of health interventions among marginalised communities.

**Aim:**

The aim of this project was to improve the community’s knowledge about hypertension by positively influencing beliefs and behaviours, leading to improved community hypertension outcomes.

**Setting:**

The study was undertaken in Ward 14, a rural area situated south-west of Gwanda District, Matebeleland South Province in Zimbabwe.

**Methods:**

We conducted a health services research utilising qualitative methods by using a community-based participatory approach using a cooperative inquiry group.

**Results:**

There was improvement in knowledge about awareness and primary prevention of hypertension. Community hypertension care was established through competence training of village health workers (VHWs) and more persons living with hypertension were enrolled into care. Pill pickup rate and treatment compliance improved and the community’s confidence in VHWs was restored. Community hypertension screening, treatment registers and health facility referrals were established.

**Conclusion:**

The community was empowered; the VHW was established as a key link between the community and the formal health delivery. This was a sustainable form of improving community hypertension health outcomes.

## Introduction

Hypertension is an important worldwide public health challenge because of its high prevalence and concomitant risks of cardiovascular and kidney diseases. The prevalence of hypertension has been on the increase because of aging and growth in world population.^1^ Hypertension is most harmful to people with comorbidities or added risk factors such as diabetes mellitus, hypercholesterolemia and tobacco use.^2^ Risk factors for hypertension include living a sedentary lifestyle, consuming diets with high fat and salt content, obesity, lack of exercise, smoking and excessive alcohol intake.^3^

Globally, cardiovascular diseases account for about 17.3 million deaths annually and a third of these deaths are linked to hypertension.^4,5^ It is estimated that by year 2030, about 23 million cardiovascular deaths will have occurred globally, with 85% of deaths occurring in low- and middle-income countries.^5^ In African communities, the challenges in managing hypertension lie in prevention, diagnosis and treatment.^6^ In Zimbabwe, the lowest prevalence of 17.9% (95% confidence interval: 17% – 19%) was recorded across three provinces in mixed study setting for both urban and rural settings.^7^

Participatory action research (PAR) is classified under implementation research, embedded within emancipatory critical paradigm and it seeks to understand the participants’ cycle of lived experiences.^8^ Participatory action research is based on reflection, data collection and action involving the researched who, in turn will take appropriate action to improve their own health.^9^ Community-based participatory research has been found to be an important tool to develop and implement health interventions among marginalised communities.^10^ It is a key strategy in translating research into action to reduce health disparities.^8,11^ Participatory action research seeks to empower and lead to people having control of their lives while ensuring community involvement in the whole research process.^8,9^

To enable success, there should be trust among the communities while intervention programmes should be sustained post project implementation.^11^ Community hypertension knowledge can be improved and sustained through the active participation of village health workers (VHWs) as a link between formal health delivery system and the community. Village health workers are trusted health workers primarily in developing countries, chosen from the community by the community or health services organisations to promote health by conducting community outreach work.^12,13^ They are trained on health promotion, common medical conditions and home-based care and are answerable to the community.^12,13^ Village health workers are supervised by the nurse in charge at the health centre.^12^ They attend regular meetings at the clinic at least once a month.

## Chronic care management model on hypertension

**FIGURE 1:**
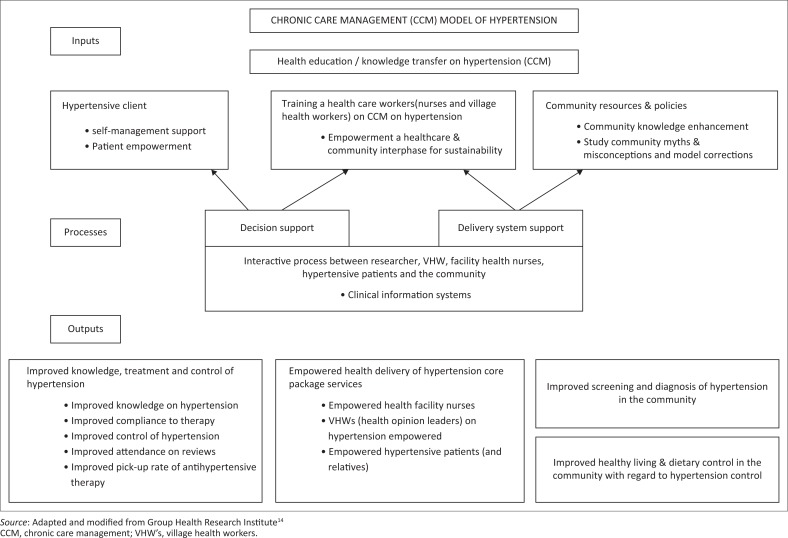
Conceptual framework on chronic care management model on hypertension.

The Donabedian model (Figure 1) identifies the inputs, processes and outputs. The model is applied to chronic care management (CCM) adapted and modified to suit hypertension awareness, treatment and control in Gwanda District. This framework is designed for improving care at both individual and population levels considering patient, healthcare provider and health system needs.^14^ Therefore, the study activities were linked to the community health needs as adapted to CCM of hypertension.

The model includes six key interdependent components: (1) community resources, (2) health system support, (3) self-management support, (4) delivery system design, (5) decision support and (6) clinical information systems.^14^ The major input is knowledge transfer on hypertension through a continuous iterative process between the researchers, community and health delivery system implementers that make up the cooperative inquiry group (CIG).

The aim of a community-based action research is to bring about change that has a positive social value. This project aimed at improving the community’s knowledge about hypertension by positively influencing beliefs and behaviours emphasising on primary prevention. It was anticipated that this approach would contribute to improved community hypertension outcomes.

## Methods

### Study design

We conducted health services research utilising qualitative methods by using a community-based participatory approach using a CIG. A PAR typically follows a number of steps: request for assistance, negotiation, planning, and action and/or implementation. The CIG members were drawn from the community and participated in conducting the research. They were involved in designing intended themes for outcomes and possible strategies to achieve the outcomes. The CIG included hypertensive patients, VHWs and community leaders who were invited or nominated from the community that was being researched. The principal investigator (PI) and clinic health workers joined the CIG as members. In this diverse group, decisions were made by consensus and power was mutually shared so that the resultant product became a shared reality.

### Study setting

The project was undertaken in Ward 14, a rural area situated about 50 km south-west of Gwanda town, Matebeleland South Province in Zimbabwe. The PI with the assistance of the community gatekeeper, the district administrator (DA), met to determine the suitability criteria for the study site, which were the following: (1) the community leaders should be agreeable to implementing the PAR; (2) the ward should have a primary health care centre; and (3) there had to be an existing health centre committee (HCC) or a community advisory board (CAB). Gwanda District population was 115 778 inhabitants, which was 16.9% of the provincial population. Ward 14 had 1384 households, 5867 inhabitants of which 55% were women. Agriculture was the economic activity that sustained community livelihood; however, recurrent droughts and poverty characterised the way of life for the communities.

### Ward 14 (Sengezane) community engagement

The DA facilitated the initial meeting with the ward councillor and the PI, which led to subsequent meetings to map a way forward. The councillor then called for a meeting with key boundary partners at the rural health centre, which was attended by 27 people comprising clinic nurses, community leaders, CAB, HCC members and patients living with hypertension (PLWHT). The scope of the study was explained, and implementation logistics were deliberated. Consenting members at the meeting were purposively invited and nominated to join the CIG.

Further community engagement was facilitated by the ward councillor as the project was introduced to the entire community during a ward meeting. The CIG coordinator, the PI and two other CIG members were afforded an opportunity to explain the concept of the project to the ward leadership and subsequently to the community in separate meetings on the day. It was agreed that identified areas of concern pertaining to hypertension be deliberated on during contact sessions (focus group discussions [FGDs]) that were held at the ward centre on that day.

### Negotiation and cooperative inquiry group setup

The CIG was made up of a total 22 persons (including the PI and two nurses from the clinic) despite initially anticipating a group of 10–15 participants. The 22 CIG members were agreed upon in anticipation of participant dropouts during the study. The CIG comprised of 8 PLWHT, 10 were VHWs and 1 was a community leader (ward councillor). A consensus to invite all consenting VHWs in Ward 14 to join the CIG was agreed upon. They then formed the backbone of the project in facilitating community-centred education and care for PLWHT. The inclusion criteria comprised a health worker involved in managing PLWHT, a VHW, a PLWHT and a community leader serving in community committees, for example, the HCC, village development committee or the CAB. The exclusion criteria for CIG enrolment were chronically unstable PLWHT who were unable to mobilise or attend CIG meetings or any invited persons who were not willing to volunteer their services to the research project.

The PI assumed the role of a ‘coach’ in leading team formation while the CIG appointed a VHW to be the CIG coordinator. The CIG agreed that the project would focus mainly on the primary prevention of hypertension as well as hypertension treatment and control in all the six villages of Ward 14. The terms of reference were agreed upon with CIG members who signed consent forms and an ethical code of practice was agreed upon. A timeline of 8 months to complete the whole project was set. At this stage, the Ministry of Health and Child Care Gwanda District Health Services and the University of KwaZulu-Natal, Discipline of Public Health Medicine were invited to be part of the project aiding with logistics and funding, respectively.

The aims and objectives of the CIG were to design acceptable and culturally appropriate interventions that would prevent and promote the control of hypertension while advocating healthy lifestyles. The CIG identified problems relating to hypertension control and generated ideas on how to solve hypertension-related problems. Data were subsequently analysed using thematic analysis, which consisted of familiarisation of data, developing initial codes, extracting themes from these codes, reviewing and renaming these and finally producing a report for the team. Skills were developed, and proposed solutions were implemented in the community. After several planning meetings and by way of consensus, the CIG agreed a 6-month period for community implementation of the CCM model (Figure 1) on hypertension care. The implementation was to be concluded by a joint community feedback meeting and a graduation ceremony for the CIG members.

### Data collection

The study was divided into two phases. Phase 1 consisted of a baseline epidemiological study where quantitative data were collected by all CIG members using interviewer-administered questionnaires, whose results will be shared in a separate paper. Phase 2 involved implementation of CCM of hypertension using a CIG. Quantitative data collected during phase 1 were used to inform the thematic aggregation of qualitative data that were used by the CIG in phase 2 of the study

### Qualitative data collection

Focus group discussions were used to assess community and VHWs knowledge gaps. There were two FGDs during ward meetings and a third FGD with VHWs. These were in the form of a guided discussion to indirectly measure knowledge while identifying gaps. Saturation of knowledge was reached when all possible basic information pertaining to hypertension primary prevention awareness and treatment had been discussed. In-depth interviews with health facility staff members, VHWs and community members, inclusive of PLWHT, were conducted to assess the knowledge and community perception on hypertension treatment and control. The PI was aware of his possible influence on the process and actively dealt with reflexivity by ensuring that decision-making power was shared equally with CIG members at all stages of the research process. The discussions were audio taped, transcribed and checked for authenticity by the PI and the CIG members. Hard copies of paper notes of the conversations were jotted down and filed.

The CIG members were trained on the World Health Organisation–CCM model (Figure 1) as applicable to the PAR methodology using creative inquiry tailored on hypertension care. This also involved monthly CIG action reflection cycles where key themes identified from phase 1 of data collection, FGDs and in-depth interviews were generated and were used to plan interventions. The CIG members did reflect on interactions they had with their clients and/or community members. A consensus on knowledge gaps that needed to be filled was reached leading to modules for training being developed by the PI as requested by CIG members.

The process of reflection and action incorporated participant observations, informal discussions, interviews and the documentation of field notes kept by individual CIG members. Each CIG member was requested to keep field notebooks where they recorded key experiences, thoughts, emotions and reactions. Abstract concepts and generalisations which emanated from this process were documented as field notes and shared during CIG meetings. Implications and testing of new knowledge, group reflection on lived experiences and consensus building were done as part of action reflection cycles. Strategies formulated and new plans to be implemented in the community and at the health facility were agreed upon. Monthly meetings for CIG members included sharing of lived experiences, reflection, consensus building and planning. Action reflection cycles were done in a stepwise thematic analysis where new strategies, ideas and knowledge obtained from the field were incorporated into subsequent action reflection cycles. All activities planned and implemented were designed by the CIG in response to new-found ideas and strategies generated and agreed during the action reflection cycle meetings.

### Chronic care management implementation trainings and strategies

The CIG after realising their knowledge deficits requested the PI to prepare training modules on selected themes in line with hypertension CCM. The CIG members would then share experiences and demonstrate new knowledge acquired during the preceding month, analyse and interpret generated data. Extensive discussions were held to assist each CIG member to tackle any challenges they faced in the field. Planning for implementation on the next monthly cycle was done while CIG members were allocated tasks to accomplish and report back. Village health workers and some CIG members were trained on practical blood pressure measurement, interpretation and were capacitated on additional basic skills in hypertension care.

### Cooperative inquiry group training modules and practical learning

Three training modules were developed primarily by the PI and were taught to the CIG members to address identified knowledge gaps. Module 1 summarised the epidemiology of hypertension, explained how to conduct community diagnosis and provided methods for using PAR methodology in hypertension CCM. Module 2 covered topics such as interpretation of blood pressure reading and cardiovascular complications of hypertension. Module 3 included skills development in using the digital blood pressure measuring device, the first-line antihypertensive medicines and the common side effects of these drugs. Practical sessions with supervision by the PI and professional nurses were conducted.

### Cooperative inquiry group hypertension clubs and door-to-door visits

A ‘CIG hypertension club’ comprising all CIG members was designed to encourage all members to get their blood pressure recorded and publicly share their recordings. Group interpretation of readings would be done and quantitative output variables such as pill pickup rate, attendance to reviews, compliance to treatment (pill counts) and blood pressure control were measured for PLWHT in the ‘club’. The model of the ‘clubs’ would be decentralised to the community revolving around the VHWs to enable community screening, peer support, improved treatment compliance and health education.

### Hypertension clinic day and community outreach

A pilot ‘hypertension day’ was organised at Sengezane clinic where the CIG mobilised patients and put into practice the knowledge they had gained. Patients who presented at the clinic were screened for high blood pressure by the VHW in the CIG supervised by the clinic nurses. The nurses prescribed medicines to those who needed them while other CIG members gave health education and counselling on hypertension. The health education sessions were delivered in the form of group discussions facilitated by CIG members. The CIG sourced additional antihypertensive medication for the clinic to cater for the anticipated increased numbers of patients while the procured digital blood pressure machines complemented the existing resources at the clinic. Subsequently, the monthly CIG meetings coincided with the hypertension clinic days.

In collaboration with Expanded Programme of Immunisation, the CIG members organised themselves and carried out a hypertension outreach. This was conducted at Ntanye village, which is about 30 km from the clinic and it was a replica of the hypertension clinic day. This was another example of a collaborative community-driven hypertension service delivery package by the CIG.

### Cooperative inquiry group monthly meetings

The findings from phase 1 and findings from planned phase 2 CCM community implementation activities formed the backbone of discussions in the CIG meeting. The CIG meeting deliberations were also guided by the issues identified during action reflection cycles. The findings were then used to come up with themes for further learning, planning and implementation in the community. Upon completion of collective reflection, planning and implementation sessions, the summary findings from the monthly meetings were aggregated as a report for the next CIG meeting by the PI. A final consensus and way forward was to be agreed by the CIG and reported to the community. The nurses at the clinic were the secretariat of the CIG; they kept adopted minutes and registers for all CIG meetings and deliberations for easy access by members.

### Trustworthiness of the study

The PI was aware of his possible influence on the process and actively dealt with reflexivity by ensuring that decision-making power was shared equally with CIG members at all stages of the research process. The research participants appreciated that all CIG members were equal; there was mutual respect as part of the code of ethics and the CIG coordinator was chosen from the community. Cooperative learning was encouraged which demystified preformed subjective ideas that the PI may have harboured. Reported data and interpretation were cross-checked and agreed upon by the research participants through a consensus process.

The quality of enquiries was assured by audio recording of all discussions and a back-up of paper notes and pictures for proceedings was kept. The PI compiled minutes for the CIG meetings; they were discussed, corrected and adopted as a corrected record by the CIG in the next meeting. Adopted minutes of the CIG meetings were kept in a file at the clinic under the care of nurses for easy access by the CIG. The discussions were transcribed and checked for authenticity by the PI and the CIG members. Triangulation of data between methods was conducted and research participants confirmed interpretation of research findings.

### Ethical considerations

All phases of this research were jointly approved by the Medical Research Council of Zimbabwe (MRCZ/A/2136) and the Biomedical Research Council of the University of KwaZulu-Natal, South Africa (BFC318/16). Permission was sought from the Ministry of Health and Child Care Zimbabwe through the Provincial Medical Director, Matebeleland South and the District Medical Officer for Gwanda to conduct the study.

## Results

### Community engagement findings

The community responded positively to the research and appreciated their lack of knowledge about hypertension. The study was dubbed ‘a developmental project in the ward’ by the councillor. The community members agreed that it was prudent for the CIG to acquire and share as much knowledge with them for improved hypertension outcomes. Repeatedly, the commonest phrase was ‘our people are dying from this disease (hypertension) due to lack of knowledge’. It was evident that almost all FGD participants believed that stress was the main cause of hypertension, and thus, hypertension was not regarded as a killer disease.

Similarly, VHWs lacked knowledge about hypertension. They were conversant with commonly used traditional herbs, although they knew about antihypertensive therapy. The VHWs conceded that they lacked clinical skills and understanding on screening, diagnosis and management of hypertension. One VHW said ‘that is the domain of the nurses, we can only refer the patients to the clinic’. Hypertensive patients also lacked appreciation of the importance of compliance to treatment and resultant complications that may arise. The commonest challenge they faced was antihypertensive medication stock-outs at the clinic. There were other challenges faced by hypertensive patients such as long walking distances to and from the clinic and stigma or pressure from the community to default medicines that were deemed to be ‘poison’. One elderly man said ‘I did not know that defaulting medication can lead to complications in the future since this disease is painless’.

### Cooperative inquiry group meetings, hypertension clinics and outreaches

**FIGURE 2:**
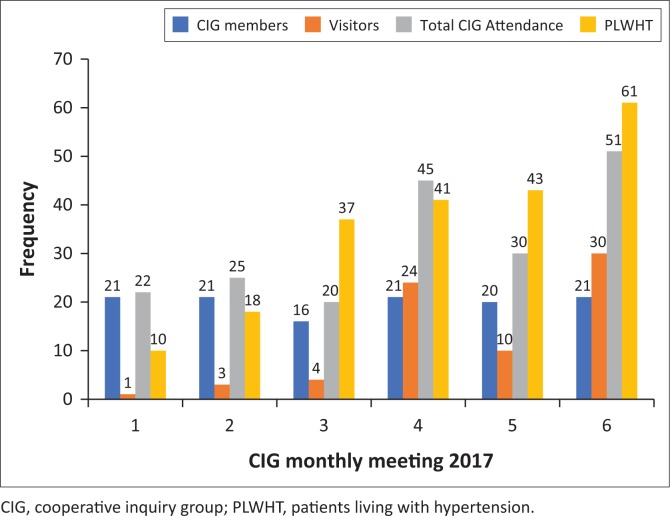
Attendance of cooperative inquiry group meetings and hypertension clinic by patients living with hypertension at Sengezane clinic, Ward 14 in Gwanda District, community-based action research project, 2017.

Six CIG action reflection cycle meetings were held and the number of visiting PLWHT (non-CIG members) who joined the CIG meetings to gain knowledge about hypertension steadily increased over the study period. The visitors to the CIG meetings increased from 1 to 30 (Figure 2). There were no dropouts among CIG members and meeting attendance remained high and satisfactory for the whole project. The number of hypertensive patients seen during the hypertension clinic day also increased from 10 to 61 between May and October 2017, respectively (Figure 2). Some PLWHT who were non-CIG members became regular CIG meeting attendees and benefited by improving their overall knowledge regarding hypertension.

A ‘CIG hypertension club’ comprised all CIG members and held six monthly meetings. During the first few meetings, nurses measured and documented blood pressure for all CIG members and with their permission the results were discussed with the group. After module 3 training, the measurement and documentation of blood pressure was done by VHWs as part of their ongoing development. The ‘CIG hypertension club’ included eight PLWHT, of whom five had poorly controlled blood pressure. They had monthly pill counts and pill pickup rates checked by their peers for 6 months and their adherence improved to 100%. One patient remained with poorly controlled hypertension after the third month and the medication was reviewed. By the fifth month of peer support group, all eight CIG members had stable blood pressures. The rest of the CIG non-hypertensive club members remained free of hypertension during the study. Source documents from the clinic and registers from the VHW were used to triangulate quantitative output data.

A total of 43 new patients from the community were diagnosed with high blood pressure during the study period. During the ‘hypertension day’, 53 patients were seen and 13 new diagnoses were made while 38 patients were seen during the ‘hypertension outreach’ at Ntanye. A total of 61 new and repeat hypertensives were recorded and handed over to the clinic nurses for follow-up on the final hypertension clinic day.

The CIG members were also assigned as hypertension advocates and also visited known PLWHT in their respective villages. They empowered the local community with their new-found knowledge. They perused the patient-held records and advised patients on compliance, shared dates of subsequent meetings and invited willing patients to attend ‘hypertension days’ at the clinic. Some volunteered to join CIG meeting and this practice was actively encouraged but they were considered as visitors.

Ten VHWs were trained on blood pressure measurement, documentation, interpretation and basic hypertension management. A monitored practical implementation post-training supervised by PI and nurses was done. ‘Community hypertension clubs’ revolving around the VHW with a strong component of peer support and community hypertension medicine refills were implemented. Using funds mobilised from the community, the CIG procured two digital blood pressure machines. The VHWs screened community members for hypertension and established and updated hypertension registers (Table 1). The PLWHT ‘club members’ who were unable to visit the clinic got their medicine refills through the VHW.

**TABLE 1:** Comparison of number of patients living with hypertension on village health workers’ registers before and after hypertension chronic care management project implementation as reported on the final cooperative inquiry group meeting; Ward 14, Gwanda District, Matabeleland South Province, Zimbabwe, 2017.

Village	Pre-CIG	Final CIG meeting
Sengezane	4	32
Nhlamba	-†	46
Paye	7	20
Ntanye	-†	37
Sizhubane	-†	22
Bethel	7	18

**Total**	**18**	**195**

CIG, cooperative inquiry group.

† , Hypertension register non-existent and hypertensive patient numbers in the village could not be verified.

At inception, three villages had no hypertension registers while those who had the registers were not updated and only 18 patients could be verified. At the final CIG meeting, VHW registers had a combined total of 195 patients (Table 1). This was a mirror reflection of patients who now had trust in the VHW as a source of a hypertension care package. A referral system was established from the community to the clinic integrating with existing VHW referral slips used primarily for maternal and child health. A positive feedback loop was established between the VHW and the clinic resulting in the clinic having updated hypertension registers as well.

A shortage of medicines was a key contributor to defaulting medicines. In case there were medicine stock-outs at the clinic, PLWHT would buy medicines from private pharmacies utilising the club’s pooled transport resources and would report to their VHW. A reconciliation, updating of clinic registers and defaulter tracking systems were piloted by VHWs working with clinic nurses within the CIG. The CIG sourced medicines from Gwanda hospital and received donations from a private pharmacy to supplement stocks at the clinic. This resultantly improved the antihypertensive medicines stocks at the clinic, thus pill pickup rate and treatment compliance for PLWHT improved during the study.

Other reasons for defaulting medicines were the misconception that, ‘treatment compliance is dangerous and the body gets used to medicines’. Irrational drug use as in sharing of medicines was noted and appropriate education was imparted. The use of traditional herbs ‘*isihaqa, fadzamhuka, izambane leganga, mupandamakore and umkhomo*’ was common because of community beliefs and fuelled by recurrent antihypertensive medicines stock-outs. Hypertension talks, CIG discussions and action reflection cycles identified several myths and misconceptions which were corrected. Some myths included beliefs that taking grounded paw-paw leaves cures blood pressure and hypertension is because of witchcraft. An example of a misconception was the belief that one could ‘feel’ when one’s blood pressure was elevated. Another misconception was the belief that by taking antihypertensive medication, one gets ‘used to the medication’ resulting in the treatment no longer working.

Varied testimonies by hypertensive patients were used as a source of learning during CIG meetings and during the community feedback meeting. One such was an 82-year-old man who started treatment in 1978 and had no complications of hypertension or from medication. He said ‘I had been taking medication for the past 40 years or so, I have never defaulted treatment and I am still going strong without any problems’. Another key testimony came from a stroke survivor who ended up with a disability of a left hemi-paresis and joint contractures because of poor treatment compliance. He said:

‘I was diagnosed of hypertension, I got naughty and defaulted treatment because I felt no pain anywhere. Look at me now, I lost my job in town and I am back in the rural areas paralyzed and unable to fend for myself.’ (Participant 1, male, 52 years old)

As the project continued, it was reported that patients started visiting the VHWs for hypertension screening, treatment and health education. The CIG coordinator said, ‘now I no longer go to their homes, they come to my house for hypertension screening’. Realising the success and benefits of the programme, the CIG decided to extend the programme to PLWHT outside of Ward 14 where willing patients could join the activities and meetings.

### Community feedback

The community feedback was attended by 157 people including health workers from various health institutions and the local Chief. There were health education messages provided by the CIG, primary school students, health clubs and PI. Testimonies were also shared by hypertensive patients who were in the CIG demonstrating knowledge that they had acquired. One CIG member said:

‘we have learnt that hypertension is a lifestyle disease, if you eat healthy, exercise, avoid alcohol and smoking while complying to antihypertensive medicines, surely you can live longer without complications from hypertension.’ (Participant 2, female, 67 years old)

Another gentleman remarked, ‘I was dead and the clinic nurses resurrected me all because I was not taking my medications’. This was after he had malignant hypertension and had acute confusional state. He was admitted and stabilised in hospital. The health education messages from the dramas and songs centred on hypertension risk factors, lifestyle factors, complications and the need to comply to medication.

The Provincial Medical Director (PMD) who was the guest of honour, representing the Ministry of Health and Child Care adopted the CIG and committed to improve the logistical supply chain challenges for antihypertensive medicines. The CIG promised to sustain the community-based hypertension care package and proposed to explore community-based healthcare packages on diabetes mellitus and prostate cancer in collaboration with Ministry of Health. The PMD handed hypertension certificates of competence to 21 CIG members at the end of the community feedback meeting.

### Final consensus building

A final consensus building noted improvements in the knowledge of the CIG members. More so, the regular PLWHT non-CIG members who visited the CIG meetings had gained substantial knowledge comparable to the CIG members. The community became involved and empowered on the development and implementation of acceptable and appropriate hypertension service delivery package. They were confident to expand their knowledge base to other prevalent non-communicable diseases such as cancer and diabetes. The VHWs had successfully been task-shifted to take up community hypertension care and be the link between the community and the formal healthcare delivery system.

## Discussion

In this study, a community was engaged in setting up, planning, implementation and interpretation of study findings. Action research requires active participation in setting the agenda, involvement in analysing situations in their communities, developing, implementing appropriate interventions and identifying how data can be disseminated to improve health in communities.^11,15^ This is important in capacity building, tapping from a wealth of community knowledge while correcting myths and misconceptions for both the researcher and the researched. We demonstrated that hypertension can successfully be managed in the communities through the participation of VHWs. These were trained in diagnosis, treatment and monitoring of patients in the community. Individuals in the community who had not been diagnosed and those who had defaulted were brought into care. Trained VHWs contribute to a positive feedback loop between the clinic and the community thus bridging the gap that existed between the formal health delivery and the community.^11^

The community was responsive to contributing money to buy digital blood pressure machines that were used by the VHWs. This was one way of harnessing community resources to benefit health delivery. The community’s confidence in VHWs as a source of health service delivery on hypertension was poor initially, but with the inception of the health clubs and clinic hypertension days, trust and confidence improved. If implemented on a population-wide basis, half of hypertension-related deaths could be averted through the adherence to antihypertensive care plans.^16^ The sustainability of community hypertension care requires an expanded role of VHWs to include non-communicable diseases (NCDs). The role of VHWs in Zimbabwe had been restricted to communicable diseases and child healthcare. It is known that community health workers with minimal medical training can be as effective as physicians in helping patients lower their blood pressure through counselling and the supervised prescription of medicines.^17^ Decentralising hypertension service delivery package into the community enables task-shifting to community screening, diagnosis, follow-up care and timely referrals to the primary health facilities. We managed to form community health clubs that assisted patients with moral support and health education. This would ultimately contribute to improved compliance to treatment and monitoring for defaulters. Lessons can be drawn from HIV care programmes in Africa and the adoption of a similar, primary care–driven models of care, which is now targeting a 90% coverage for antiretroviral therapy of affected populations.^18^

The CIG members who were hypertensive benefited from the training sessions in that they realised the importance of compliance to treatment and this information filtered to the community. There was no attrition in the CIG and this can be an indicator that the content of the programme was useful to the participants. Indices such as pill pickup rate, compliance and blood pressure control improved during the study. This was because of improved knowledge by the patients and also improved availability of medication. Evidently, there was poor knowledge regarding hypertension and weak community health education platforms. This was confirmed by an active role for alternative medicine in managing hypertension that influenced the social norms on health. Also, the community had low economic status, which negatively influenced health-seeking behaviour. This was a rural disadvantaged community where there is little money to spend on health and a low literacy rate. Therefore, hypertension service delivery packages need to address these factors because ignoring them fails to holistically treat a patient while missing opportunities for controlling cardiovascular disease in the most vulnerable communities.^2^ There is need for a robust community engagement strategy to influence a positive social change.

We noted that the local clinic experienced medicine stock-outs prior to the study inception and we managed to stabilise availability of medicines during the study period. The patients had developed trust in the health delivery system for service provision and the numbers who attended the clinic for blood pressure treatment increased. Recurrent stock-outs of antihypertensive medicines at the clinic contributed to a lack of community’s confidence with public health delivery system’s commitment to hypertension care. Making available thiazides can close the treatment gap for less than US$20.00 per person per year.^16^ Programmes to fight NCDs like hypertension are markedly underfunded and despite NCDs being the leading cause of death in adults worldwide, they have struggled to attract sufficient funding.^2,16,19^ Therefore, investment in hypertension treatment can and does have a large, sustained impact at a low cost.

During final consensus building, it was noted that the level of knowledge among CIG members on hypertension had markedly improved and non-CIG members who attended meetings regularly and had gained substantial knowledge. At the end of the project, the empowered community realised the benefits of action research and promised to sustain community-based hypertension care and expand this to the management of diabetes mellitus and prostate cancer. Therefore, knowledge transfer is possible and effective in communities where there is low formal education for the benefit of communities to take control of their health. This can be a window for the expansion of NCDs care into the community. Using this approach, value beliefs and practices of an ethnic or racial group within a particular community can influence acceptance and adherence to health messages.^11^

The PAR was implemented in one administrative ward and thus the findings cannot be generalised to the other districts or provinces which may have a different setting. However, findings can be transferrable to another setting. The study findings can be used to inform the district health authorities on cross-cutting priorities and issues to consider on sustainability of hypertension management within rural communities. This project managed to highlight a number of implications for health services: (1) the feasibility of engaging the communities and encouraging community participation on an equal power sharing; (2) the value of integrating community and health service programmes as this created a buy-in from all the boundary partners; (3) the importance of empowerment on the success and future sustainability of community-based health-related programmes. Ultimately, this will enable the crafting of appropriate community-approved and long-term sustainable service delivery packages.

## Conclusion

There was a successful engagement between the community, health workers and the researcher in a PAR project leading to improved awareness on hypertension primary prevention, treatment and control. We demonstrated knowledge transfer that led to a positive community behavioural change among PLWHT, their families and the community. Importantly, the engagement of all boundary partners and community participation created ownership of the project, trust and cooperation with health workers. The participation of nurses enhanced their understanding of the sociocultural factors as a determinant of health and helped ensure future sustainability.

This study proved that it was possible to empower VHWs with self-confidence and appropriate hypertension care competency skills. Valuable cooperative learning occurred among researchers, health workers and managers in understanding the sociocultural determinants and practices within the communities they work in. Community members had an opportunity to actively participate and decide how service delivery packages are crafted for their own benefit.
